# Uncovering food purchase behaviors and their shifts using Hidden Markov Modeling

**DOI:** 10.1186/s12966-026-01963-4

**Published:** 2026-08-07

**Authors:** Anna M. Ruokolahti, Haniyeh Danesh Doost, Anna-Leena Vuorinen, Henna Vepsäläinen, Reija Autio, Maijaliisa Erkkola, Mikael Fogelholm, Hannu Saarijärvi, Jaakko Nevalainen

**Affiliations:** 1 Health Sciences Unit, Faculty of Social Sciences, Tampere University, Tampere, Finland; 2 Department of Food and Nutrition, University of Helsinki, Helsinki, Finland; 3 Department of Clinical Chemistry, Fimlab Laboratories, and Finnish Cardiovascular Research Center - Tampere, Faculty of Medicine and Health Technology, Tampere University, Tampere, Finland; 4 Faculty of Management and Business, Tampere University, Tampere, Finland

**Keywords:** Transaction data, Loyalty card data, Grocery purchase data, Supermarket data, Big data, Dietary assessment, Transition models, Purchase patterns

## Abstract

**Background:**

Investigating food purchase data is a novel and effective approach to large-scale dietary assessment, but many traditional analysis methods often overlook the temporal structure of food purchases. This study demonstrates a data-driven approach for identifying and describing changes in food purchasing behaviors over time, using Hidden Markov Modeling (HMM) to assess the underlying structure of individual-level time-series purchase data.

**Methods:**

The loyalty cardholders of the LoCard study provided access to purchase data from 9/2016 to 12/2018 and responded to a background questionnaire providing cross-sectional information on sociodemographic characteristics and self-estimated share of purchases from the retailer (purchase loyalty) (*n* = 29,245). Food group-level data of participants with ≥ 40% purchase loyalty to the retailer were aggregated monthly, subjected to a dimension reduction, and then an HMM was fitted on the monthly data. Food purchase behaviors (i.e. modeled states) were labeled, and their estimated prevalence and transition probabilities across behaviors were examined by gender, age, household structure, and household income.

**Results:**

Nine food purchase behaviors were grouped as health-oriented: (1) ‘balanced’ (2) ‘home cook’, (3) ‘flexitarian’, and (4) ‘planetary’; discretionary-oriented: (5) ‘traditional’, (6) ‘convenience’, (7) ‘sugar-sweetened beverages and snacks’, (8) ‘beer, sweets, and treats’; or baby-oriented: (9) ‘baby formula and foods’. Participants’ food purchase behaviors remained relatively stable, as demonstrated by low month-to-month transition probabilities (< 5%). Compared to other age and household categories, younger adults and households with two adults and children were most likely to change food purchase behaviors. Transitions typically occurred within the health-oriented or discretionary-oriented groups, or away from the baby-oriented group. Women, older adults, and households without children more often exhibited overall health-oriented behaviors, while younger adults specifically leaned towards plant-based purchasing behaviors, such as ‘flexitarian’ and ‘planetary’.

**Conclusions:**

HMMs offer an interpretable approach for analyzing longitudinal food purchase data collected at high frequency over extended periods, having an ability to capture temporal changes in purchasing behaviors over time. The identified food purchase behaviors differed across sociodemographic characteristics, revealing distinct and relatively stable purchasing behaviors. Trends in their prevalences were only gradual, and transitions took place mainly between similar purchase behaviors, which highlights that purchase behavior is not easily altered.

**Supplementary Information:**

The online version contains supplementary material available at https://doi.org/10.1186/s12966-026-01963-4.

## Background

Monitoring dietary behaviors is crucial for identifying nutritional differences within and between populations and for understanding population health, as well as monitoring and evaluating diet-related recommendations and public health policies. Traditionally, national nutrition surveys assess food consumption using self-reported methods, such as food frequency questionnaires, food records, and recalls [1, 2]. However, in addition to being resource-consuming to implement, such approaches are prone to biases and misreporting, and they often reflect delayed snapshots of dietary behavior collected over multi-year intervals [3]. In contrast, food purchase data collected through retailers’ loyalty programs offer a valuable addition by capturing the types of foods bought in households, providing a proxy for food and beverage consumption. In lieu of absolute amounts of food (and beverage) consumed, purchases can better capture the proportion of different foods bought. Nevertheless, purchase data has shown acceptable concordance with self-reported dietary intake [4, 5] and allows for more timely, lower-burden monitoring, making it a high-potential tool to assess both individual and population-level food purchase habits [6, 7].

Individual dietary habits and food purchases are similarly shaped by a complex interplay of sociodemographic, environmental, and intrinsic factors [8]. Prior research has demonstrated associations between food purchasing patterns and characteristics such as age, gender, household structure, education, geographic location, population-level events (e.g. change in policy, pandemic, recessions), and consumer attitudes and motivations [9–14]. Describing both differences in food purchase behaviors and longitudinal shifts in these behaviors can be beneficial for designing nutritional messaging to individuals and sub-populations, examining associations with health and environmental outcomes, and advancing research on the determinants of purchase behavior. Consequently, identifying objective and comprehensive data sources is essential for accurately characterizing how food purchases vary and evolve over time.

Food purchase data from loyalty cards is particularly valuable in research for its potential to provide detailed, objective longitudinal data on household consumption habits. Loyalty card data refers to the digital records generated when consumers use store-issued cards or mobile phone applications during purchases, allowing tracking of individual purchases over time. This passive, large-scale data collection reduces reliance on smaller-scale self-reported information, which is subject to recall and social desirability biases, as well as reporting error [15, 16]. The detailed structure of loyalty card data typically contains time, product, and quantity of purchases (e.g. price, quantity, weight) over a period of time, along with the cardholder’s pseudoidentity that enables linkage with other individual-level data [17]. These data can be further linked to external data sources such as food composition databases and with questionnaire data collected within the same study. The latter ideally includes information about the degree of food purchase loyalty and household structure. However, leveraging these data comes with the challenge of managing high-dimensional individual-level time-series data, where the volume and variability of information across and within individuals, time points, and product categories require advanced analytical approaches [18].

Traditional principal component analysis and factor analysis have previously been used to study purchase behaviors [14, 19–21]. These techniques are useful for simplifying multivariate datasets by grouping similar patterns or reducing redundancy and noise. However, they typically rely on aggregating data over a defined study period into a single observation per individual, which effectively removes the temporal information inherent in purchase data. As a result, these methods overlook the dynamic nature of food choices over time, and do not capture within-person transitions. These transitions may be critical elements when attempting to understand behavioral shifts due to factors, like policy changes, interventions, trends, or health diagnosis, as well as assessing individual or subpopulation potential readiness to shift behavior.

In contrast, Hidden Markov Models (HMMs) offer a more nuanced approach by enabling researchers to identify latent “states”, which can be interpreted as patterns or clusters between which individuals move, and to examine how individuals transition between these states over time [22]. Food purchase data can be temporally aggregated as desired (e.g. annually, monthly, or weekly per individual). This allows a more comprehensive analysis of longitudinal data, uncovering how both individual food purchasing behavior evolve, and potentially revealing drivers of these shifts. However, applying HMMs to such high-volume data necessitates careful preprocessing of data to ensure appropriate modeling and interpretation [23]. In sum, HMMs present a promising, data-driven alternative for modeling hidden structures in loyalty card data, introducing possibilities for predicting future purchase behavior, identifying consumers who are more susceptible to behavioral shifts following, e.g., nutrition policies or other steering methods, such as taxation of foods or dietary guidelines, and understanding the likelihood and directionality of those changes. Nevertheless, though HMM has been utilized in many different fields [22], the application of HMMs in the context of food purchase data remains underexplored to date. To support researchers in adopting methods suited for high-dimensional, temporal data, this study aims to provide a detailed methodological framework for the application of HMMs to food purchase data. It demonstrates what new insights HMMs can yield, such as illustrating transitions in behavior, observing transition probabilities, and investigating the temporal evolution of behavior prevalences. Furthermore, the study also aims to show how HMM results can be used in describing sociodemographic differences in food purchasing behavior among Finnish households.

## Methods

### Study population, design, and data collection

The purchase data of 47,066 primary cardholders were obtained from loyalty members (≥ 18 years old) of the S Group who consented to participate in the LoCard Study. The S Group is the leading grocery store retailer throughout Finland, holding 47% market share in 2017, and sharing majority (80–90%) of the total market with only two other leading retailers [24]. The primary cardholder was invited, via email from the retailer, to participate in the study by providing informed consent to retrospectively share purchase data from September 2016 until December 2018. Additionally, consenting participants were provided with an online questionnaire from June to August 2018 to gather additional household and background information that would be linked to their purchase data. The study was reviewed by the University of Helsinki Ethical Review Board in the humanities and social and behavioral sciences (Statement #21/2018).

The retailer provided participant information on gender and birth year. Birth year was then used to calculate participants’ ages at the time of the questionnaire data collection and categorized by age group in decades as previously presented [25]. In total, 36,526 (78%) participants responded to the questionnaire. In order to capture a sample of participants with representative coverage of all food purchases, only participants who reported ≥ 40% of their household’s total food purchases to the retailer (i.e. purchase loyalty) were included in the sample (*n* = 29,573, 63%) [25]. Participants reported the number of individuals living in their household and specified their age groups: 0–6 years, 7–17 years, 18–24 years, 25–64 years, and 65 years or older. Based on this information, each household was categorized into one of five family structure types: single-adult households, one adult with child(ren), two adults, two adults with child(ren), or other (including households with three or more adults or those with an unclassified family structure) [5]. Household gross income was reported on a 7-point Likert scale, with categories: < 1500, 1500–2999, 3000–4499, 4500–5999, 6000–7499, 7500–8999, ≥ 9000 €/month. For each household, the midpoint of the reported income category was used as an estimate of monthly income, which was then adjusted for household size by dividing by the square root of the total number of household members, in accordance with the OECD square root equivalence scale [26]. The resulting OECD-adjusted household income was further categorized into groups of < 1,000 €, 1000–1999, 2000–2999, 3000–3999, and ≥ 4000 €/month.

Purchase data was categorized by food and beverage groups according to the health-centered classification described previously [27]. The initial product groups were matched with corresponding foods and beverages from the Finnish Institute for Health and Welfare Fineli food composition database to obtain energy and nutrient contents for each group [28]. In brief, the data consisted of 161 groups identified based on nutritional value and nutrient content (e.g. cereals, breads, and flours divided by fiber content, dairy food groups by fat content, beverages by sugar content, alcoholic beverages as percentage of alcohol). All food and beverage purchase variables were converted to grams per 2500 kcal (10.5 MJ) to account for varying household sizes and loyalty. To assess the healthfulness of each food purchase behavior, they were compared against the 2014 Finnish national food-based dietary guidelines [29].

If no purchases in a food or beverage product group were made at a given month, the observation was set to zero, and if no purchases at all were made for the given month, the observation was set as missing. For each participant, data was then summed monthly for the 28 consecutive months (from 09/2016 to 12/2018). Furthermore, to ensure that shopping adequately reflected overall food purchase habits, incomplete months were excluded if food and beverage purchases totaled less than 17,500 kcal, corresponding to one week of intake at 2500 kcal per day. Roughly 63% (*n* = 29,245) of study participants had available questionnaire information and sufficient purchase data available for at least one month, resulting in 694,753 valid monthly purchases to analyze.

Compared to excluded participants (i.e. those who did not answer the questionnaire (*n* = 10,540), reported having lower than 40% purchase loyalty to the S Group (*n* = 6953), or had inadequate minimum purchases (i.e. all months missing or incomplete, *n* = 259), those included in the study sample were more likely to be women. Among participants who completed the questionnaire, those included in the analysis were more likely to live in two adult households with child(ren). No differences were seen in age or income level.

### Data preparation and dimension reduction analysis

To identify relevant food and beverage product groups for analysis, data reduction was performed using both theory-driven (d = 36 food group variables removed) and data-driven (d = 46 food group variables removed, d = 66 food group variables removed all together; see Supplemental Table 1 for details) approaches. Theory-driven approach eliminated food group variables considered irrelevant based on their nutritional relevance as assessed by nutrition researchers. For the data-driven reduction, purchase data were first winsorized at the 1st and 99th percentiles to moderate the effect of outliers, and food group variables with a purchase volume variance greater than 10 were retained. The purchase data were then log-transformed and standardized to unit standard deviation to address skewness, and to prevent foods commonly consumed in larger quantities from disproportionately influencing the results due to their weight or volume.

Next, to reduce the number of food group variables analyzed to a smaller set of dimensions informative for the HMM, while still preserving as much of the original information as possible, an adaptive dimensional reduction (ADR) was performed on the dataset initially reduced using the combined theory- and data-driven approaches. ADR is an iterative dimension reduction approach, which was designed to estimate a subspace for improved clustering performance over that offered by standard methods like PCA [30]. While PCA identifies directions of maximum variance, these directions do not necessarily align with those that best separate natural clusters. In this context, the resulting cluster structures are important as they provide the foundation from which the hidden states of the HMM are derived. ADR addresses this by initializing with PCA, then performing k-means clustering in the projected subspace, and next updating the projection based on the cluster centroids and followed by a singular value decomposition [30]. Lastly, the latter two steps are repeated until convergence [30]. The ADR coordinates were estimated using a randomly selected, independent observation from each individual to satisfy its assumptions, and the same estimated coordinates were then computed on the full dataset.

### Hidden Markov Modeling for individual-level food purchase data

HMMs are probabilistic models used to infer an unobserved sequence of states based on observed data. A Markov process is followed, meaning that the probability of transitioning to a new state depends only on the current state instead of the full sequence of preceding states. In addition to state transitions, HMMs define an emission probability for each state, which quantifies the likelihood of observing a specific data point given that the individual is in that state. These emission distributions must be specified or estimated as part of model construction. In our application, the observations (i.e. purchase data) are represented by multivariate ADR dimensions, which define the emission probability distribution. A multivariate normal distribution with uncorrelated components was assumed, consistent with the properties of ADR, and incomplete sequences (i.e. missing observations) were treated as missing at random.

The msm package in R provides a framework for fitting continuous-time multistate models to longitudinal data, where individuals transition between a set of hidden states over time [31]. However, incorporating too many parameters can lead to interpretability and convergence issues. Initial values for model parameters were set to simple values (see analytic code [32]). Model fitting was carried out using the default optimization algorithm; the Broyden–Fletcher–Goldfarb–Shanno algorithm [31].

The number of ADR dimensions (2-5) was initially assessed for a subset of roughly 10% of participants (*n* = 3000), selected to reduce the computational burden and processing time, by comparing hidden states (intended to represent food purchase behaviors) to food purchases. Four and five dimensions provided states that were distinct and representative of clear and interpretable food purchase behaviors. Within four and five ADR dimensions, the number of states (2-10) was compared with Bayesian Information Criteria (see Supplemental figure 1), which considers maximum log likelihood while penalizing for the number of model parameters, with a heavier penalty for larger data sets. Subsequently, a model using five dimensions and nine states was implemented to obtain individual state sequences, overall state prevalences and transition probabilities between them.

As a sensitivity analysis, participants who reported 81–100% purchase loyalty to S Group were included in the modeling (*n* = 14,048 participants with 352,654 monthly observations), with little variation in modeled states and transition probabilities from the main analysis (data not included). Another sensitivity analysis was conducted using a revised exclusion criterion of less than 14,000 kcal (2000 kcal per day over seven days) for monthly inclusion, which resulted in the inclusion of an additional 69 participants and 20,909 monthly observations. The resulting states were similar but, as expected, less distinct than those in the present findings and were therefore not shown.

For comparisons of food purchase behaviors and transition probabilities across sociodemographic groups, differences were assessed using chi-squared and Bonferroni-adjusted pairwise proportion comparisons for independent observations, and generalized linear mixed-effect models with random intercepts for observations that were not independent, with Tukey-adjusted post-hoc comparisons. Transition probability matrices were reported for monthly and annual (12-month) intervals, with annual probabilities derived from the estimated transition rates using all 28-months of data. Sequence index plots were used to illustrate the estimated monthly states and transitions by gender, age categories, household structure, and income level. To aid visual comparison, only participants with ≥ 24 months of data were included in the main findings (see Supplemental Figures 3–6 for full sample). All statistical analyses were conducted with R version 4.4.3 [33]. Analytic code for our HMM implementation is freely available online [32].

## Results

The study included 29,245 participants and 694,753 monthly purchase observations. On average, participants had 23.8 (SD 6.6) months of data (i.e. months with adequate food purchases), with 47% (*n* = 13,602) having data for all 28 months, and 70% (*n* = 20,690) having at least 24 months of data over the 28-month study period. Participants under 30-years-old (12%), over 70 years old (9%), and men (34%) were less represented in the study population (Table 1). Roughly half of the participants (49%) reported over 80% purchase loyalty to the retailer for food purchases.

**Table 1:** Background information on the participants of the study (*n* = 29,245)

	n (%)	mean (SD)
Age categorized (years)		
< 30	3592 (12)	25.3 (2.9)
30–39	6240 (21)	34.7 (2.9)
40–49	5963 (20)	44.3 (2.9)
50–59	5797 (20)	54.3 (2.8)
60–69	4944 (17)	64.5 (2.8)
≥ 70	2709 (9)	74.0 (3.9)
Missing	0 (0)	
Gender		
Women	19,423 (66)	
Men	9822 (34)	
Missing	0 (0)	
Household income^1^ (€/month)		
< 1000	2530 (9)	683 (128)
1000–1999	4348 (15)	1545 (238)
2000–2999	8454 (29)	2434 (210)
3000–3999	6493 (22)	3582 (278)
≥ 4000	5447 (18)	5230 (928)
Missing	1973 (7)	
Household structure		
Single adult	7527 (26)	
One adult and child(ren)	1343 (5)	
Two adults, no children	10,774 (37)	
Two adults with child(ren)	6836 (23)	
Other^2^	2633 (9)	
Missing	132 (0)	
Food retailer loyalty (%)		
41–60	6072 (21)	
61–80	9125 (31)	
≥ 81	14,048 (48)	
Missing	0 (0)	

^1^Household income OECD-adjusted

^2^includes households with ≥ 3 adults or those with an unclassified family structure

Each cell shows the deviation of the mean of actual food purchases of those observations inferred to belong to the state from the grand mean across all observations (in SD of the food group). Darker shades of blue/red indicate deviations closer to ± 1 SD

**Fig. 1:**
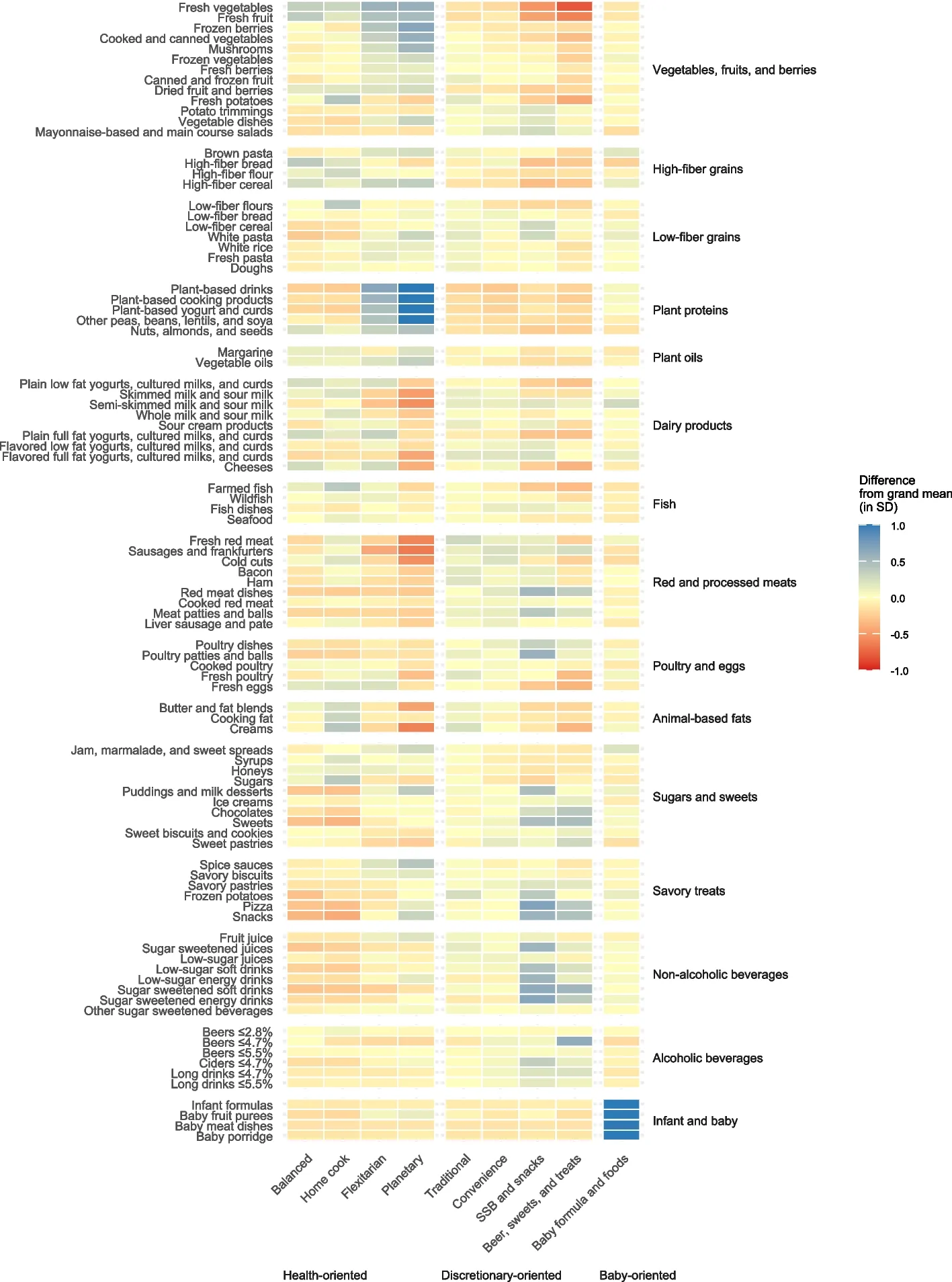
Heatmap of mean food purchases by estimated food purchase behavior relative to overall grand mean

Overall, nine distinct food purchase behaviors (previously referred to as "states") were identified from the data and labeled by nutrition researchers based on their characteristic purchase profiles, including the relative frequency of different food groups and overall nutritional quality. To facilitate interpretation, these states were categorized into three overarching behavioral groups based on similarities in their nutritional profiles: health-oriented, discretionary-oriented, and baby-oriented food purchase behaviors. Figure 1 illustrates the deviations from the grand mean (in standard deviations of the food group) in food purchases across each behavioral state.

The health-oriented group was characterized by lower purchases of red and processed meats and sugar-sweetened beverages (SSBs), and a greater emphasis on plant-based products. This group included the states labeled as ‘balanced’, ‘home cook’, ‘flexitarian’, and ‘planetary’. The ‘balanced’ state reflected higher than average purchases of fresh fruits, vegetables, and high-fiber grains, and lower purchases of, for example, snacks and SSBs. ‘Home cook’ behaviors were consistent with those of a home cook or baker, showing distinct features compared to the ‘balanced’, including purchases of butter, cooking fats, creams, fresh potatoes, sugars, and low-fiber flours, while still favoring fresh vegetables and high-fiber grains over more processed options. ‘Flexitarian’ behavior emphasized a broad range of plant-based foods, including vegetables, fruits, and berries, with both dairy and dairy alternatives, while limiting red and processed meat purchases. ‘Planetary’ behavior demonstrated the most plant-centric state, with relatively low purchases of all animal-based products, including dairy.

In contrast, discretionary-oriented food purchase behaviors were marked by higher purchases of red and processed meats, fewer plant-based foods, and lower intake of high-fiber grains. Food purchase behaviors such as ‘traditional’ and ‘convenience’ shared similarities, though ‘convenience’ exhibited higher purchases of ready-made dishes and salads, sweet and savory pastries, and alcoholic beverages. The ‘SSB and snacks’ purchase behavior was defined by particularly high purchases of SSBs, low-sugar soft and energy drinks, sugary alcoholic beverages, pizza, snacks, meat patties and balls, and red meat dishes. The ‘beers, sweets, and treats’ showed elevated purchases of beer, SSBs, pizza, snacks, sweets, and chocolates.

Finally, the ‘baby formula and foods’ behavior stood apart as the sole representative of baby-oriented grouping. This food purchase behavior was primarily defined by high purchases of infant formula and baby food products, likely to reflect the shared needs of households with infants, despite otherwise varied food purchasing habits.

**Fig. 2:**
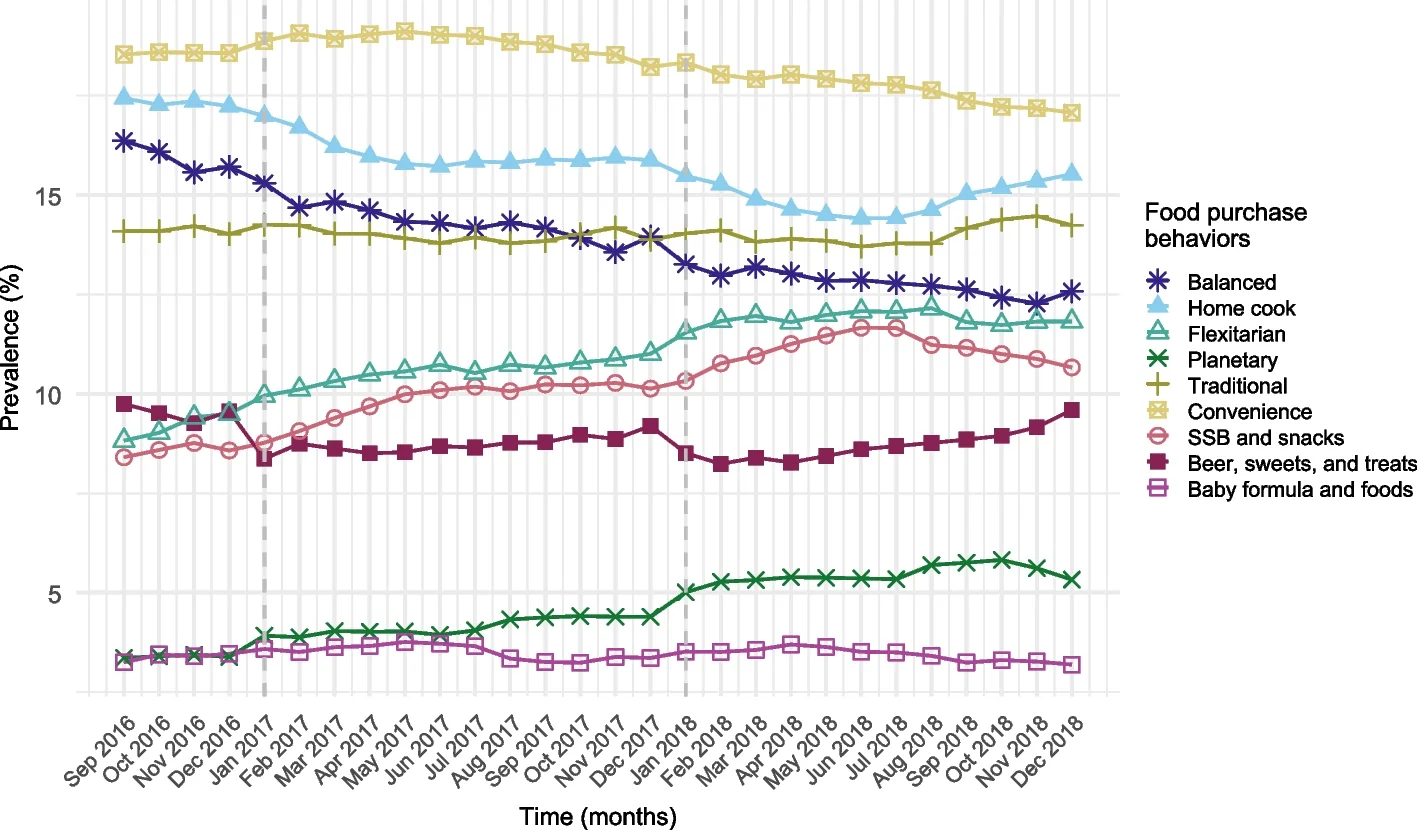
Prevalence of food purchase behaviors over from 9/2016–12/2018 in study participants (*n* = 29,245). SSB: Sugar-sweetened beverages

Prevalences of food purchase behaviors ranged between 3–19% over the study period (Fig. 2). The most prevalent states included ‘convenience’ and ‘traditional’ from the discretionary-oriented behaviors, and ‘home cook’ and ‘balanced’ from health-oriented behaviors. The lowest prevalence was found in the baby-oriented behavior; ‘baby formula and foods’. In Fig. 2, the ‘flexitarian’, ‘planetary’, and ‘SSB and snacks’ behaviors seemed to steadily but modestly increase in prevalence over the 28-month study period. Overall, strong seasonal trends were not observed in most behaviors, with the exceptions of ‘home cook’, which consistently became less popular during the summer months, and ‘beers, sweets, and treats’, which generally declined at the very start and peaked at the end of each year.

Moreover, each food purchase behavior was compared with food-based dietary guidelines (Supplemental Table 2). Health-oriented food purchase behaviors all estimated to, on average, meet recommendations for red meat consumption (< 100 g raw meat/day/10 MJ (2400 kcal)), and both ‘planetary’ and ‘flexitarian’ behaviors additionally met national recommendations for fruit and vegetable consumption (edible amount minimum of 500 g/day/10 MJ (2400 kcal)) [29]. Conversely, none of the discretionary-oriented behaviors met fruit and vegetable recommendations, and all but ‘beers, sweets, and treats’ exceeded the recommended limit for red meat consumption. Notably, no food purchase behavior met recommendations for fish (minimum of 100 g cooked fish 2 times/week) or nuts and seeds (30 g per day).

Participants tended to maintain highly consistent food purchase behaviors on a month-to-month basis (Table 2). Though transitions were similar whether assessed monthly or yearly, slightly more fluctuation was observed in annual purchases. Except for the baby-oriented behavior (9.6%), the estimated probability of staying in the same behaviors remained relatively high over yearly periods (41–69%), suggesting high overall consistency in participants' purchasing habits (Supplemental Table 3). Nevertheless, some transitions between different food purchase behaviors occurred at higher probabilities than others. For instance, participants categorized under the 'planetary' behavior had a higher chance of transitioning to the 'flexitarian' behavior, and vice versa. Moreover, those exhibiting 'flexitarian' behavior showed elevated transition probabilities toward ‘balanced’ behavior within the health-oriented group, indicating a progression of movement between related purchase behaviors. In general, transition probabilities between behaviors were highest among similar food purchase behaviors, i.e., within their respective groups of health-oriented and discretionary-oriented.

**Table 2:** Monthly transition probabilities between food purchase behaviors from study participants (*n* = 29,245)

		Health-oriented				Discretionary-oriented				Baby-oriented
		Balanced	Home cook	Flexitarian	Planetary	Traditional	Convenience	SSB and snacks	Beer, sweets, and treats	Baby formula and foods
Health-oriented	Balanced	**0.955**	0.012	0.015	< 0.001	< 0.001	0.007	< 0.001	0.007	0.005
	Home cook	0.009	**0.969**	0.007	< 0.001	0.006	0.006	< 0.001	< 0.001	0.003
	Flexitarian	0.013	0.006	**0.925**	0.028	0.004	0.005	0.002	0.003	0.014
	Planetary	0.001	0.001	0.052	**0.925**	0.001	0.001	0.005	0.008	0.007
Discretionary-oriented	Traditional	< 0.001	0.004	0.004	< 0.001	**0.968**	0.007	0.009	0.001	0.006
	Convenience	0.003	0.005	0.005	< 0.001	0.008	**0.943**	0.008	0.020	0.008
	SSB and snacks	< 0.001	< 0.001	0.002	0.003	0.010	0.010	**0.948**	0.018	0.008
	Beer, sweets, and treats	0.009	0.001	0.004	0.004	0.001	0.036	0.020	**0.919**	0.007
Baby-oriented	Baby formula and foods	0.019	0.011	0.045	0.010	0.026	0.044	0.026	0.020	**0.799**

Rows represent the starting state in a given month, and columns represent the state in the following month. *SSB* Sugar-sweetened beverages. Diagonal values indicate the estimated probability of staying in the same state, while off-diagonal values indicate the probability of transitioning to a different state. The largest transition probability in each row is bolded

**Fig. 3:**
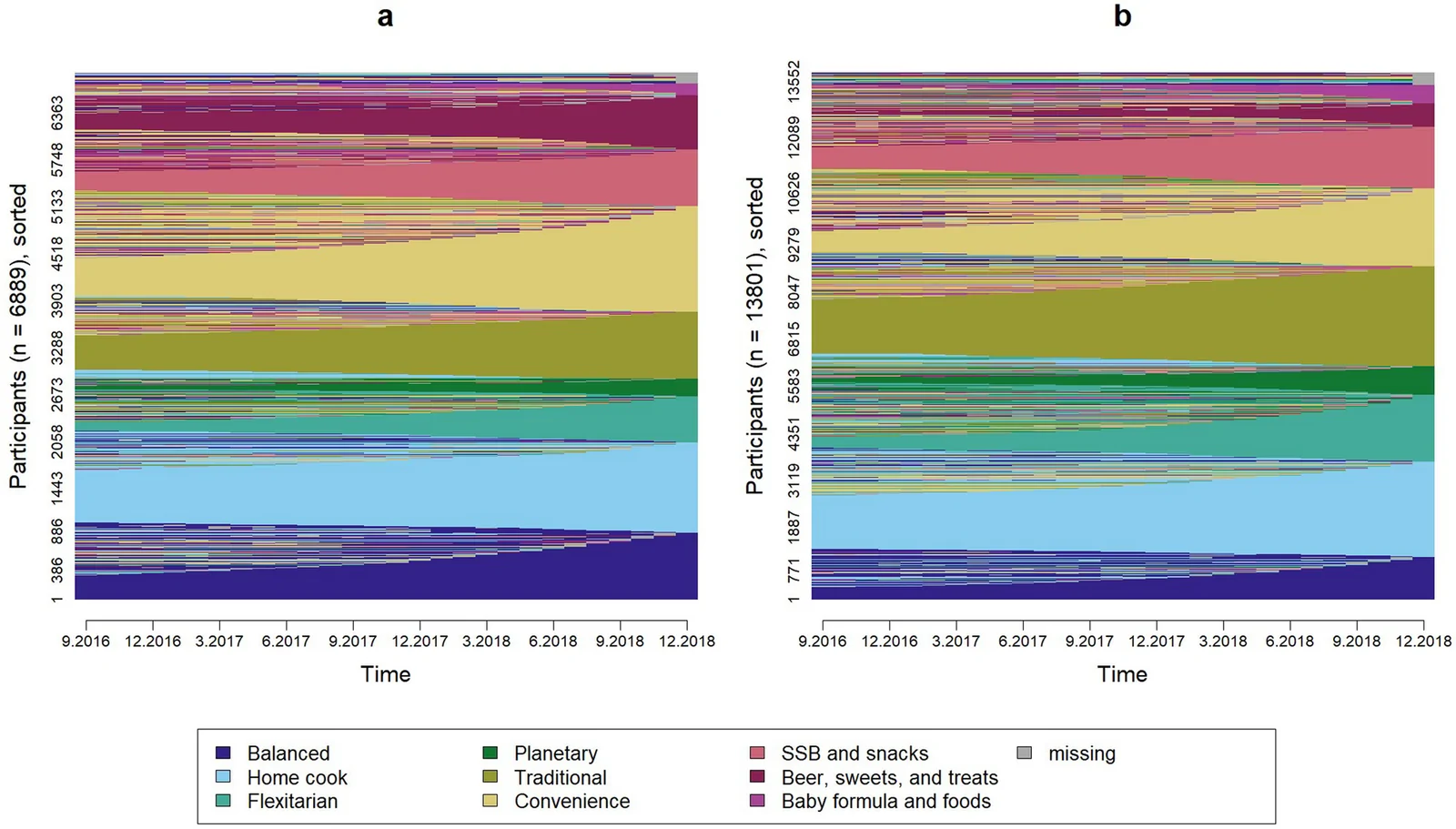
Food purchase behaviors by gender in participants with ≥ 24-months of data (*n* = 20,690). Sequence index plots show estimated monthly states and transitions for participants from 9/2016–12/2018 grouped by gender sorted by last food purchase behavior (state) observed. SSB: Sugar-sweetened beverages. **a** Men, **b**. Women

**Fig. 4:**
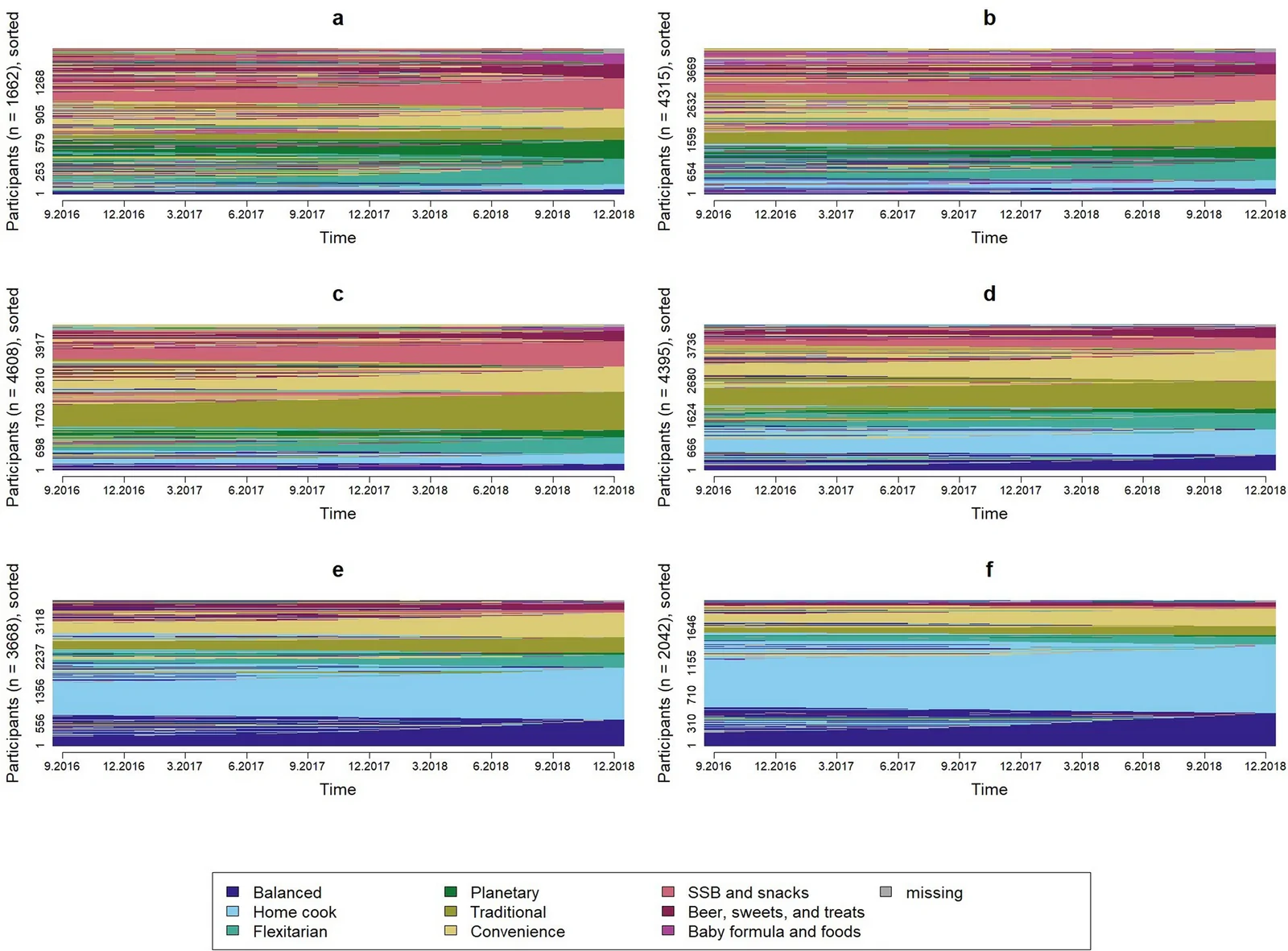
Food purchase behaviors by age in participants with ≥ 24-months of data (*n* = 20,690). Sequence index plots show estimated monthly states and transitions for participants from 9 9/2016–12/2018 grouped by decade-categorized age sorted by last food purchase behavior (state) observed. SSB: Sugar-sweetened beverages. **a**. Under 30 years old, **b**. 30–39 year old, **c**. 40–49 year-old, **d**. 50–59 year-old, **e**. 60–69 year-old, **f**. 70 year-old and older

**Fig. 5:**
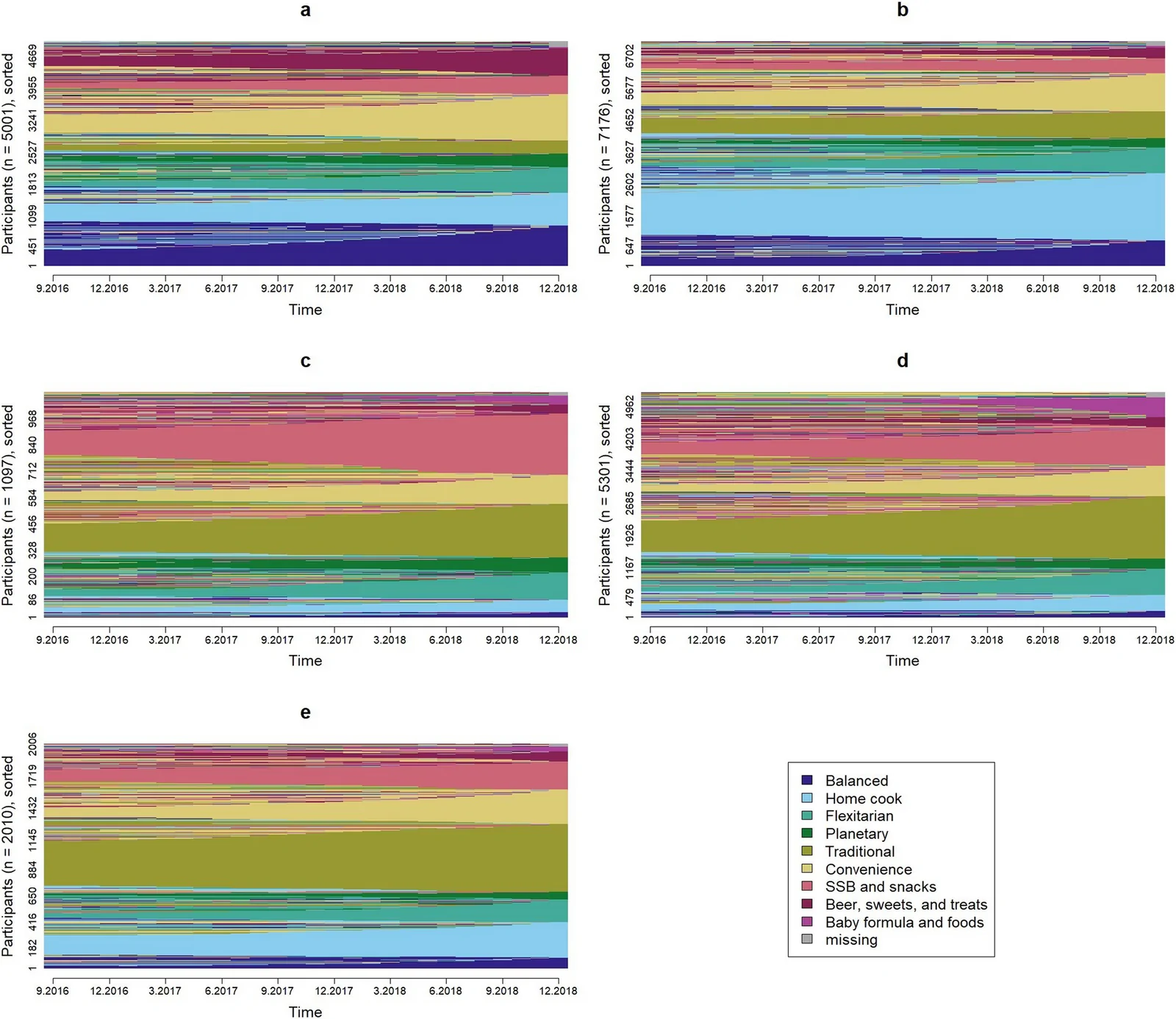
Food purchase behaviors by household structure in participants with ≥ 24-months of data (*n* = 20,690). Sequence index plots show estimated monthly states and transitions for participants from 9/2016–12/2018 grouped by categorized household structures sorted by last food purchase behavior (state) observed. SSB: Sugar-sweetened beverages. **a**. One adult households, **b**. Two adult households, **c**. One adult households with child(ren), **d**. Two adult households with child(ren), **e**. Other, including households with three or more adults or those with an unclassified family structure

**Fig. 6:**
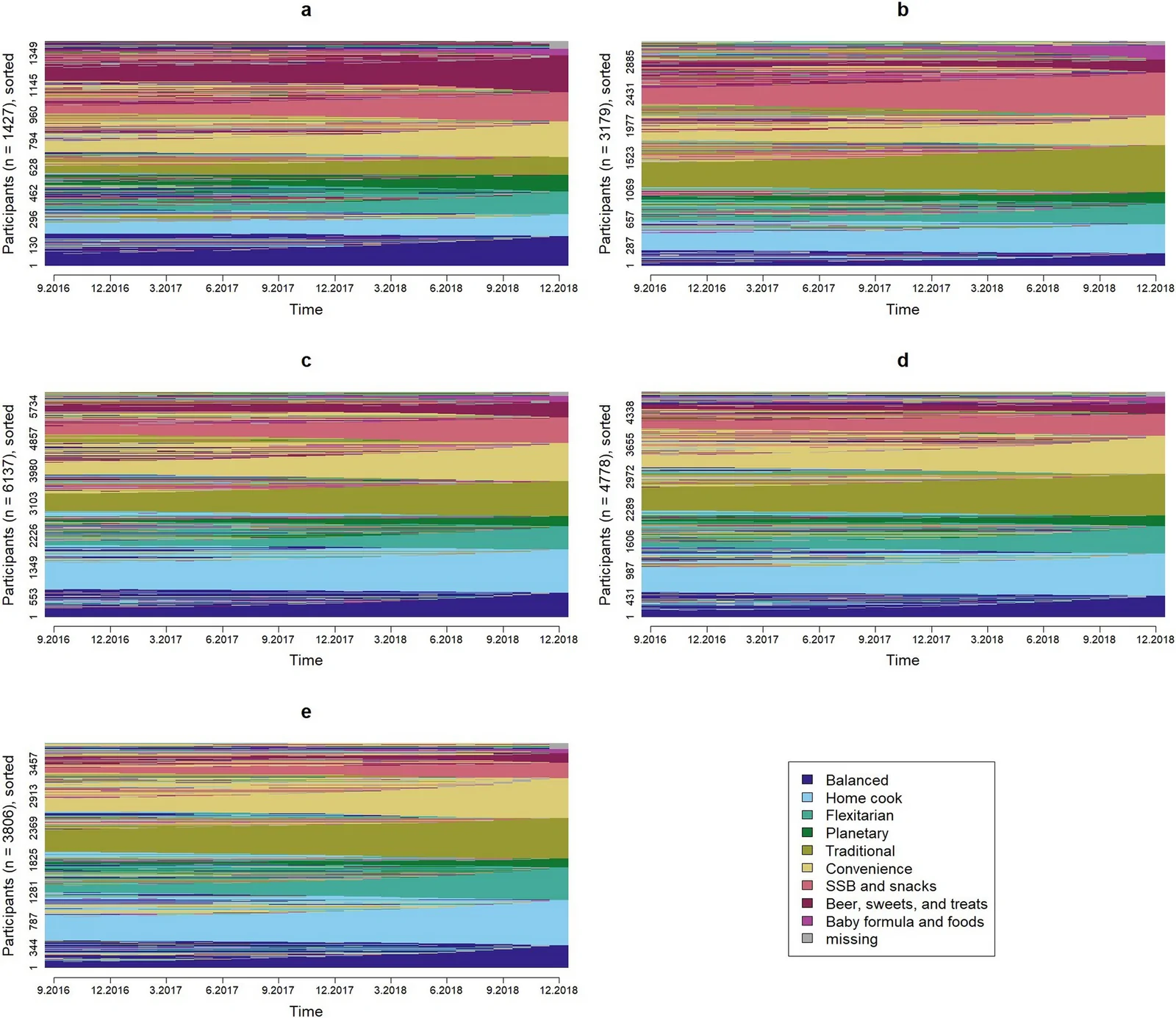
Food purchase behaviors by household income in participants with ≥ 24-months of data (*n* = 20,690). Sequence index plots show estimated monthly states and transitions for participants from 9/2016–12/2018 grouped by categorized OECD-adjusted household income levels sorted by last food purchase behavior (state) observed. SSB: Sugar-sweetened beverages. **a**. < 1000 euros/month, **b**. 1000–1999 euros/month, **c**. 2000–2999 euros/month, **d**. 3000–3999 euros/month, **e**. ≥ 4000 euros/month

Figures 3, 4, 5, and 6 illustrate the transitions of participants by gender, age, household structure, and household income for participants with at least 24 months of data. While roughly half (47%, *n* = 13,810) of participants stayed within one food purchase behavior throughout 28-month study period, those who transitioned between behaviors tended to transition between two (35%, *n* = 10,327) or three (13%, *n* = 3893) behaviors, with only 4% (*n* = 1215) transitioning between more than three different food purchase behaviors. The latter participants were more likely to be women, under forty years old, or with children in the household.

Participants’ gender, age, household structure, and income all significantly differed between health, discretionary, and baby-oriented food purchase behaviors, when assessed using modal food purchase behavior. Women tended to have more health-oriented (48% vs. 43%, *p* < 0.001) and slightly more baby-oriented purchase states (2.7% vs. 2.0%, *p* < 0.001) than men, whereas men were often more discretionary-oriented in their food purchases than women (55% vs. 49%, *p* < 0.001). Participants aged 60 and over favored health-oriented food behaviors, like ‘balanced’ and ‘home cook’, whereas younger age categories exhibited more preference specifically for ‘flexitarian’ and ‘planetary’ food purchase behaviors alongside increased discretionary-oriented food behaviors (Supplemental figure 2). Compared to all other participants, those aged 40–49 had the least health-oriented and most discretionary-oriented behaviors (*p* < 0.001). Moreover, health-oriented food purchase behaviors were more common among adult-only households (*p* < 0.001), specifically 59% in two-adult and 52% in single-adult households. In contrast, discretionary-oriented behaviors were most prevalent among households with children (*p* < 0.001), particularly in households with one adult and child(ren) (70%). Health-oriented behaviors were more frequent in the highest income group (54% in ≥ 4000 €/month vs. 36% in 1000–1999 €/month, *p* < 0.0001), whereas discretionary-oriented behaviors were less frequent (45% in ≥ 4000 €/month vs. 60% in 1000–1999 €/month, *p* < 0.0001). Additionally, 55% of participants in the lowest income group had discretionary-oriented behaviors, most commonly ‘beer, sweets, and treats’ (20%, see Supplemental figure 2). Baby-oriented food purchase behaviors were relatively uncommon overall but were least frequent among the participants in highest (≥ 4000 €/month) income group (1%), and most frequent in the 1000–1999 €/month income group (5%).

Overall, women had slightly more transitions than men (mean (SD): 1.16 (1.54) vs. 0.97 (1.43), *p* ≤ 0.001) and higher transition probabilities (5.1% vs. 4.3%, *p* ≤ 0.001). Comparisons across age categories indicated that all groups differed significantly by number of transitions and transition probabilities (*p* ≤ 0.01). Both the number of distinct shifts between behaviors and mean transition probabilities were inversely associated with age, ranging from the highest among participants under 30 years (1.49 (1.60) and 7.8%, respectively) to the lowest in those over 70 years (0.43 (0.88) and 1.9%, respectively). Likewise, there were more transitions on average among participants with households including children than those without. The transition probabilities ranged from 3.9 to 7.3% in different household structures, with the highest belonging to two adult households with children (7.3%). Compared to other income groups, those with an income of 1000–1999 €/month had the highest number of transitions (1.4 (1.7), *p* < 0.001). Nevertheless, all income groups had fairly similar transition probabilities (4.4–5.9%).

## Discussion

This study demonstrated how an HMM can be effectively applied to food purchase data to uncover meaningful behavioral patterns. Nine distinct, data-driven food purchase behaviors were identified, and their transition probabilities were described over the study period. The food purchase behaviors were interpreted and categorized into overarching groups: health-oriented, discretionary-oriented, and baby-oriented. Overall, food purchase behaviors were relatively stable among loyalty card holders, with 95% of participants transitioning between 0 and 3 different food purchase behaviors, and older populations exhibiting the greatest stability in consumer patterns. Moreover, women, older adults (≥ 60 years), households without children, and highest income households were more likely to exhibit health-oriented purchasing states, though younger populations favored specifically plant-based food purchase behaviors (i.e. ‘flexitarian’ and ‘planetary’). Monthly transition probabilities suggested a stepwise progression, with consumers more likely to shift directly within either the health-oriented or discretionary-oriented groups rather than between them.

Unlike traditional time-static methods, such as standard cluster, PCA or factor analysis, HMMs offer a dynamic approach that captures transitions between latent behavioral states over time that allow for the estimation of transition probabilities and the temporal evolution of food purchasing behavior. This temporal perspective provides novel observations on how individuals (or households) can transition between different food purchase behaviors, offering a more detailed understanding of consumer purchases and potential consumption behaviors, though it requires longitudinal data with sufficient measurement points and frequency to reliably capture these transitions.

The application of HMMs to loyalty card data represents an advancement over previous studies, which have largely focused on specific food groups or isolated purchase behaviors rather than modeling overall purchasing patterns [10, 34–36]. Moreover, past research has typically stratified analyses by sociodemographic factors without explicitly investigating how different groups change behaviors over time [14]. The approach utilized in this study not only allows for such subgroup comparisons, but also reveals the probabilistic nature of behavioral change, which can be beneficial for designing targeted nutrition interventions and potentially understanding causes in changing food purchase trends, as well as dietary habits.

Despite these advancements, commonalities were observed with previous loyalty card studies. For instance, a Canadian study examining the co-purchase associations of sodas, fresh fruits and fresh vegetables from 2015–2017, reported that sodas were often purchased alongside salty snacks and sweets, whereas fruits and vegetables were more commonly associated with premade salads [34]. Another study from the US describing household produce purchases from 2016–2018, found that older populations bought produce more frequently and consistently compared to younger populations [10]. Although the current findings are based on household purchase data rather than individual consumption, they still mirror national dietary survey data from Finland: women purchased more plant-based foods than men, and age differences were also evident, with older individuals purchasing more fresh fruits, vegetables, and high-fiber foods [37]. Likewise, as only 14% of men and 22% of women in Finland met fruit and vegetable recommendations in 2017 [37], it was unsurprising that most food purchase behaviors also did not meet these recommendations.

A strength of this study is the use of individual-level data covering all loyalty card purchase occasions for more than two years, which enabled temporal analyses. Furthermore, food groups were classified and assessed for nutritional quality [27], and the data have been previously validated against food frequency questionnaires [5]. In addition, background information from questionnaires allowed for further sub-group analyses. This study also introduced a powerful, yet interpretable, HMM framework for identifying and tracking transitions between hidden states over time, making them particularly well-suited for analyzing evolving food purchase behaviors from food purchase data. Though still novel in nutrition research, HMMs are fairly accessible and adaptable for use in longitudinal purchase datasets. Furthermore, the use of loyalty card data provides a passive measure of food purchasing behavior, free from the self-report bias typically associated with self-reported purchase or dietary assessments.

Despite the relatively large sample, participation in the LoCard study was limited to a small proportion of primary loyalty cardholders, where women and participants with higher levels of education were overrepresented compared to the national population [25], which may influence the population-level prevalence of certain states. Additionally, as with other methods, the lack of ground truth limits the ability to verify whether the model identifies the correct states. Nevertheless, HMMs are a powerful tool for pattern discovery and data mining, and their temporal structure can support causal inference studies when integrated with longitudinal data. The performance and interpretability may also vary depending on the structure, size, quality, and coverage of the available data. For instance, assuming that loyalty card purchases serve as a good proxy for dietary intake may be valid in the Finnish context, where S Group holds the largest market share, but may not hold in countries or retail settings with more fragmented market shares. Furthermore, it is important to acknowledge that loyalty card purchases likely reflect household-level rather than individual-level food preferences, since food purchases are often made for the entire household. As the data only captured purchases made within a single grocery store retailer, some household food purchases were not observed, which may limit the completeness of the purchasing data. However, given that the analyses utilized relative rather than absolute purchase volumes, and that previous validation work has shown these purchase records to provide a reasonably representative picture of participants’ dietary intake [5], we do not expect the unobserved purchases to substantially bias the identified purchasing behaviors at the monthly aggregation level. This interpretation was further supported by a sensitivity analysis, which only examined participants who reported the highest purchase loyalty to the retailer without meaningful changes in the model outcome. Future studies should attempt to account for temporary promotional campaigns or retailer-specific discounts, as prices are important motives of food purchase behaviors [11]. Furthermore, the HMM approach used in this study could be utilized to examine food purchase behaviors from different perspectives, such as seasonal or holiday-related variation or sub-group specific behaviors. For instance, since the baby-oriented behavior was dominant to infant formula and baby foods, there is opportunity to further investigate how the transition into parenthood influences broader household food purchasing behaviors and whether these changes vary according to household size and age of household children. In addition, to more accurately examine how participant characteristics relate to food purchase behaviors, future studies should incorporate longitudinally administered questionnaires. Lastly, the labeling of food purchase behaviors involves a degree of subjectivity and cultural context, which could affect how states are interpreted across different populations.

## Conclusions

This study highlights the potential of HMMs for uncovering food purchase behaviors in longitudinal food purchase data. By capturing transitions between behavioral states over time, HMMs provide novel, data-driven description of food purchase behaviors that offer more than traditional time-static analyses. With the growing use of food purchase data in academic research [7], it is relevant to introduce and promote optimal methods for researchers to apply. While more research is needed to clarify the biases and limitations of analytical approaches applied to purchase data, HMMs present promising opportunities for advancing the study of food purchasing behaviors.

In this study, participants generally exhibited stable food purchasing behaviors, although differences in prevalence and transitions were observed across age, gender, household structure, and income level. Participants with health-oriented food purchase behaviors were more likely to transition to other health-oriented behaviors, while those with discretionary-oriented behaviors tended to remain within that group. Overall, changes occurred slowly and in a stepwise manner, mainly between similar food purchase behaviors, indicating that these behaviors are relatively resistant to change. However, certain groups, such as young adults and two adult households with children, were most likely to change their food purchasing behaviors.

Future studies can focus on capturing and understanding potential drivers of the transitions in food purchase behaviors, such as perceptions and motivations. Additionally, if linked with longitudinal data sources, HMMs could be extended to help model disease outcomes, or to serve as predictive tools for future food purchases or to identify populations that may be more susceptible to certain behavioral shifts or responses to policies or trends.

## Supplementary Information

Supplementary Material 1.

## Data Availability

Due to the commercial nature of the data used in this research, it is not possible for data to be published alongside the manuscript. Analytic code for modeling is provided at [32].

## Abbreviations

ADR: Adaptive dimensional reduction
HMM: Hidden Markov model
OECD: Organisation for Economic Co-operation and Development
SSB: Sugar-sweetened beverages
