# Supplementary material for: Uncovering food purchase behaviors and their shifts using Hidden Markov Modeling
Source: Int J Behav Nutr Phys Act. 2026 Aug 7;23:108. doi: 10.1186/s12966-026-01963-4 (PMC13746725; doi:10.1186/s12966-026-01963-4)
Supplement: Supplementary file 1 [file 12966_2026_1963_MOESM1_ESM.docx]

**Supplemental Materials**

**Supplemental Table 1.** Food purchase data reduced from LoCard food classification^1^ for data analysis.

| Reduction step | All initial food groups | Theory-driven^2^ | Data-driven^3^ | Combined theory and data-driven^4^ |
| --- | --- | --- | --- | --- |
| Number of food and beverage products | d=161 | d=125 | d=115 | d=95 |
| Vegetables fruits and berries | | | | |
| 55 | Fresh vegetables | Fresh vegetables | Fresh vegetables | Fresh vegetables |
| 31 | Fresh fruit | Fresh fruit | Fresh fruit | Fresh fruit |
| 1 | Frozen berries | Frozen berries | Frozen berries | Frozen berries |
| 47 | Cooked and canned vegetables | Cooked and canned vegetables | Cooked and canned vegetables | Cooked and canned vegetables |
| 8 | Mushrooms | Mushrooms | Mushrooms | Mushrooms |
| 6 | Frozen vegetables | Frozen vegetables | Frozen vegetables | Frozen vegetables |
| 11 | Fresh berries | Fresh berries | Fresh berries | Fresh berries |
| 17 | Canned and frozen fruit | Canned and frozen fruit | Canned and frozen fruit | Canned and frozen fruit |
| 18 | Dried fruit and berries | Dried fruit and berries | Dried fruit and berries | Dried fruit and berries |
| 9 | Fresh potatoes | Fresh potatoes | Fresh potatoes | Fresh potatoes |
| 5 | Potato trimmings | Potato trimmings | Potato trimmings | Potato trimmings |
| 24 | Vegetable dishes | Vegetable dishes | Vegetable dishes | Vegetable dishes |
| 16 | Mayonnaise-based and main course salads | Mayonnaise-based and main course salads | Mayonnaise-based and main course salads | Mayonnaise-based and main course salads |
| 6 | Fruit, berry, and nut mixes | Fruit, berry, and nut mixes |  |  |
| High-fiber grains | | | | |
| 5 | Brown pasta | Brown pasta | Brown pasta | Brown pasta |
| 23 | High-fiber bread | High-fiber bread | High-fiber bread | High-fiber bread |
| 16 | High-fiber flour | High-fiber flours | High-fiber flours | High-fiber flours |
| 28 | High-fiber cereals | High-fiber cereals | High-fiber cereals | High-fiber cereals |
| 1 | Brown rice | Brown rice |  |  |
| 1 | High-fiber grain blends | High-fiber grain blends |  |  |
| Low-fiber grains | | | | |
| 18 | Low-fiber flours | Low-fiber flours | Low-fiber flours | Low-fiber flours |
| 53 | Low-fiber bread | Low-fiber bread | Low-fiber bread | Low-fiber bread |
| 14 | Low-fiber cereals | Low-fiber cereals | Low-fiber cereals | Low-fiber cereals |
| 29 | White pasta | White pasta | White pasta | White pasta |
| 8 | White rice | White rice | White rice | White rice |
| 5 | Fresh pasta | Fresh pasta | Fresh pasta | Fresh pasta |
| 6 | Doughs | Doughs | Doughs | Doughs |
| 1 | Uncategorized grain blends |  |  |  |
| Plant proteins | | | | |
| 9 | Plant-based drinks | Plant-based drinks | Plant-based drinks | Plant-based drinks |
| 3 | Plant-based cooking products | Plant-based cooking products | Plant-based cooking products | Plant-based cooking products |
| 7 | Plant-based yogurt and curds | Plant-based yogurt and curds | Plant-based yogurt and curds | Plant-based yogurt and curds |
| 157 | Other peas, beans, lentils, and soya | Other peas, beans, lentils, and soya | Other peas, beans, lentils, and soya | Other peas, beans, lentils, and soya |
| 20 | Nuts, almonds, and seeds | Nuts, almonds, and seeds | Nuts, almonds, and seeds | Nuts, almonds, and seeds |
| 1 | Plant-based ice creams | Plant-based ice creams |  |  |
| 2 | Plant-based puddings | Plant-based puddings |  |  |
| 4 | Other fungal protein products | Other fungal protein products |  |  |
| 20 | Vegetarian sausages | Vegetarian sausages |  |  |
| 22 | Non-dairy cheeses | Nondairy cheeses |  |  |
| 10 | Other wheat protein products | Other wheat protein products |  |  |
| Plant oils | | | | |
| 3 | Margarine | Margarine | Margarine | Margarine |
| 11 | Vegetable oils | Vegetable oils | Vegetable oils | Vegetable oils |
| Milks, yogurts, and cheeses | | | | |
| 7 | Skimmed milk and sour milk | Skimmed milk and sour milk | Skimmed milk and sour milk | Skimmed milk and sour milk |
| 10 | Semi-skimmed milk and sour milk | Semi-skimmed milk and sour milk | Semi-skimmed milk and sour milk | Semi-skimmed milk and sour milk |
| 5 | Whole milk and sour milk | Whole milk and sour milk | Whole milk and sour milk | Whole milk and sour milk |
| 5 | Sour cream products | Sour cream products | Sour cream products | Sour cream products |
| 12 | Plain low fat yogurts, cultured milks, and curds | Plain low fat yogurts, cultured milks, and curds | Plain low fat yogurts, cultured milks, and curds | Plain low fat yogurts, cultured milks, and curds |
| 20 | Plain full fat yogurts, cultured milks, and curds | Plain full fat yogurts, cultured milks, and curds | Plain full fat yogurts, cultured milks, and curds | Plain full fat yogurts, cultured milks, and curds |
| 16 | Flavored low fat yogurts, cultured milks, and curds | Flavored low fat yogurts, cultured milks, and curds | Flavored low fat yogurts, cultured milks, and curds | Flavored low fat yogurts, cultured milks, and curds |
| 40 | Flavored full fat yogurts, cultured milks, and curds | Flavored full fat yogurts, cultured milks, and curds | Flavored full fat yogurts, cultured milks, and curds | Flavored full fat yogurts, cultured milks, and curds |
| 87 | Cheeses | Cheeses | Cheeses | Cheeses |
| 2 | Powdered milks and creams | Powdered milks and creams |  |  |
| 9 | Other milks and sour milks |  | Other milks and sour milks |  |
| 9 | Uncategorized yogurts, cultured milks, and curds |  | Uncategorized yogurts, cultured milks, and curds |  |
| Fish | | | | |
| 43 | Farmed fish | Farmed fish | Farmed fish | Farmed fish |
| 54 | Wildfish | Wildfish | Wildfish | Wildfish |
| 19 | Fish dishes | Fish dishes | Fish dishes | Fish dishes |
| 22 | Seafood | Seafood | Seafood | Seafood |
| 13 | Uncategorized fish |  | Uncategorized fish |  |
| Red and processed meat | | | | |
| 161 | Fresh red meat | Fresh red meat | Fresh red meat | Fresh red meat |
| 83 | Sausages and frankfurters | Sausages and frankfurters | Sausages and frankfurters | Sausages and frankfurters |
| 22 | Cold cuts | Cold cuts | Cold cuts | Cold cuts |
| 2 | Bacon | Bacon | Bacon | Bacon |
| 10 | Ham | Ham | Ham | Ham |
| 39 | Red meat dishes | Red meat dishes | Red meat dishes | Red meat dishes |
| 27 | Cooked red meat | Cooked red meat | Cooked red meat | Cooked red meat |
| 20 | Meat patties and balls | Meat patties and balls | Meat patties and balls | Meat patties and balls |
| 12 | Liver sausage and pate | Liver sausage and pate | Liver sausage and pate | Liver sausage and pate |
| 2 | Meat jellies | Meat jellies |  |  |
| 21 | Offals | Offals |  |  |
| 7 | Canned meat | Canned meat |  |  |
| Poultry and eggs | | | | |
| 68 | Fresh poultry | Fresh poultry | Fresh poultry | Fresh poultry |
| 22 | Cooked poultry | Cooked poultry | Cooked poultry | Cooked poultry |
| 11 | Poultry patties and balls | Poultry patties and balls | Poultry patties and balls | Poultry patties and balls |
| 20 | Poultry dishes | Poultry dishes | Poultry dishes | Poultry dishes |
| 6 | Fresh eggs | Fresh eggs | Fresh eggs | Fresh eggs |
| 3 | Egg products |  |  |  |
| Animal-based fats | | | | |
| 5 | Butter and fat blends | Butter and fat blends | Butter and fat blends | Butter and fat blends |
| 3 | Cooking fat | Cooking fat | Cooking fat | Cooking fat |
| 10 | Creams | Creams | Creams | Creams |
| Sugars and sweets | | | | |
| 18 | Jam, marmalade, and sweet spreads | Jam, marmalade, and sweet spreads | Jam, marmalade, and sweet spreads | Jam, marmalade, and sweet spreads |
| 3 | Syrups | Syrups | Syrups | Syrups |
| 10 | Honeys | Honeys | Honeys | Honeys |
| 4 | Sugars | Sugars | Sugars | Sugars |
| 10 | Puddings and milk desserts | Puddings and milk desserts | Puddings and milk desserts | Puddings and milk desserts |
| 18 | Ice creams | Ice creams | Ice creams | Ice creams |
| 30 | Chocolates | Chocolates | Chocolates | Chocolates |
| 78 | Sweets | Sweets | Sweets | Sweets |
| 32 | Sweet biscuits and cookies | Sweet biscuits and cookies | Sweet biscuits and cookies | Sweet biscuits and cookies |
| 68 | Sweet pastries | Sweet pastries | Sweet pastries | Sweet pastries |
| 11 | Coated nuts | Coated nuts |  |  |
| 26 | Desserts |  | Desserts |  |
| 3 | Artificial sweeteners | Artificial sweeteners |  |  |
| 4 | Cocoa |  | Cocoa |  |
| Savory treats | | | | |
| 22 | Spice sauces | Spice sauces | Spice sauces | Spice sauces |
| 7 | Savoury biscuits | Savoury biscuits | Savoury biscuits | Savoury biscuits |
| 47 | Savoury pastries | Savoury pastries | Savoury pastries | Savoury pastries |
| 10 | Frozen potatoes | Frozen potatoes | Frozen potatoes | Frozen potatoes |
| 13 | Pizza | Pizza | Pizza | Pizza |
| 26 | Snacks | Snacks | Snacks | Snacks |
| 14 | Snack foods |  | Snack foods |  |
| Non-alcoholic beverages | | | | |
| 43 | Fruit juice | Fruit juice | Fruit juice | Fruit juice |
| 38 | Sugar sweetened juice | Sugar sweetened juice | Sugar sweetened juice | Sugar sweetened juice |
| 12 | Low sugar juices | Low sugar juices | Low sugar juices | Low sugar juices |
| 93 | Low sugar soft drinks | Low sugar soft drinks | Low sugar soft drinks | Low sugar soft drinks |
| 13 | Low sugar energy drinks | Low sugar energy drinks | Low sugar energy drinks | Low sugar energy drinks |
| 103 | Sugar sweetened soft drinks | Sugar sweetened soft drinks | Sugar sweetened soft drinks | Sugar sweetened soft drinks |
| 16 | Sugar sweetened energy drinks | Sugar sweetened energy drinks | Sugar sweetened energy drinks | Sugar sweetened energy drinks |
| 14 | Other sugar sweetened beverages | Other sugar sweetened beverages | Other sugar sweetened beverages | Other sugar sweetened beverages |
| 3 | Other low sugar beverages | Other low sugar beverages |  |  |
| 16 | Artificially sweet water |  |  |  |
| 38 | Naturally sweetened water |  | Naturally sweetened water |  |
| 61 | Unsweetened water |  | Unsweetened water |  |
| 20 | Coffee |  | Coffee |  |
| 14 | Tea |  |  |  |
| Alcoholic beverages | | | | |
| 19 | Beers max 028 | Beers max 028 | Beers max 028 | Beers max 028 |
| 116 | Beers max 047 | Beers max 047 | Beers max 047 | Beers max 047 |
| 52 | Beers max 055 | Beers max 055 | Beers max 055 | Beers max 055 |
| 93 | Ciders max 047 | Ciders max 047 | Ciders max 047 | Ciders max 047 |
| 68 | Long drinks max 047 | Long drinks max 047 | Long drinks max 047 | Long drinks max 047 |
| 26 | Long drinks max 055 | Long drinks max 055 | Long drinks max 055 | Long drinks max 055 |
| 23 | Beers max 035 | Beers max 035 |  |  |
| 12 | Ciders max 012 | Ciders max 012 |  |  |
| 3 | Ciders max 028 | Ciders max 028 |  |  |
| 9 | Ciders max 055 | Ciders max 055 |  |  |
| 5 | Long drinks max 012 | Long drinks max 012 |  |  |
| 5 | Long drinks max 028 | Long drinks max 028 |  |  |
| 1 | Other alcoholic beverages max 028 | Other alcoholic beverages max 028 |  |  |
| 1 | Other alcoholic beverages max 055 | Other alcoholic beverages max 055 |  |  |
| 7 | Wines max 012 | Wines max 012 |  |  |
| 2 | Wines max 028 | Wines max 028 |  |  |
| 10 | Wines max 055 | Wines max 055 |  |  |
| 34 | Non-alcoholic beers |  | Non-alcoholic beers |  |
| 18 | Non-alcoholic ciders |  |  |  |
| 3 | Non-alcoholic long drinks |  |  |  |
| 11 | Non-alcoholic wines |  |  |  |
| Infant and baby | | | | |
| 13 | Infant formulas | Infant formulas | Infant formulas | Infant formulas |
| 16 | Baby meat dishes | Baby meat dishes | Baby meat dishes | Baby meat dishes |
| 21 | Baby porridges and gruels | Baby porridges and gruels | Baby porridges and gruels | Baby porridges and gruels |
| 26 | Baby fruit and berry purée | Baby fruit and berry purée | Baby fruit and berry purée | Baby fruit and berry purée |
| 7 | Baby poultry dishes | Baby poultry dishes |  |  |
| 1 | Baby uncategorized dishes | Baby uncategorized dishes |  |  |
| 7 | Baby vegetable dishes | Baby vegetable dishes |  |  |
| 9 | Baby yogurt and curd | Baby yogurt and curd |  |  |
| 7 | Baby fish dishes | Baby fish dishes |  |  |
| 5 | Other baby snack |  |  |  |
| Miscellaneous |  |  |  |  |
| 26 | Baking products |  | Baking products |  |
| 19 | Chewing gum |  |  |  |
| 84 | Dietary supplements |  | Dietary supplements |  |
| 37 | Dry spices |  |  |  |
| 8 | Ketchup and mustard |  | Ketchup and mustard |  |
| 5 | Mayonnaises |  | Mayonnaises |  |
| 34 | Meal ingredients |  | Meal ingredients |  |
| 38 | Miscellaneous |  | Miscellaneous |  |
| 7 | Other sauces |  | Other sauces |  |
| 1 | Preservatives |  |  |  |
| 3 | Salad dressings |  | Salad dressings |  |
| 5 | Salts |  | Salts |  |
| 4 | Spice mixes |  |  |  |
| 3 | Stock products |  |  |  |
| 7 | Vinegars |  | Vinegars |  |
| 1 | Uncategorized |  | Uncategorized |  |

1. Food group classified from LoCard Food Classification Category 3 (27) 2.Theory-driven removes irrelevant food and beverage purchase categories. 3. Variables with low variance removed. 4. Approach used for data analysis.

**Supplemental table 2.** Approximate adherence of food purchase behaviors (mean (SD)) to the Finnish national food-based dietary guidelines^1^

| Purchased foods | Finnish recommendations^2^ | Health-oriented | | | | Discretionary-oriented | | | | Baby-oriented |
| --- | --- | --- | --- | --- | --- | --- | --- | --- | --- | --- |
|  | g/day/2500 kcal | Balanced  g/2500 kcal | Home cook  g/2500 kcal | Flexitarian  g/2500 kcal | Planetary  g/2500 kcal | Traditional  g/2500 kcal | Convenience  g/2500 kcal | SSB and Snacks  g/2500 kcal | Beer, Sweets, and Treats  g/2500 kcal | Baby formula and foods  g/2500 kcal |
| vegetables, fruit, and berries^3^ | >523 | 482  (299) | 449  (227) | 562  (271) | 610  (317) | 342  (168) | 340  (208) | 239  (157) | 163  (168) | 351 (215) |
| red + processed meat^4^ | <105 | 72.4  (70.8) | 94.9  (60.1) | 59.4  (55.0) | 32.3 (45.3) | 113.9 (55.3) | 111.8  (71.1) | 122.4 (65.1) | 101.8  (83.0) | 89.7  (58.2) |
| nuts and seeds | 31 | 5.8 (13.5) | 4.5  (9.6) | 6.8  (12.5) | 7.8 (14.0) | 2.3  (5.8) | 2.2  (7.5) | 1.2 (4.7) | 1.5 (7.4) | 2.3 (7.1) |
| alcohol^5^ | <11-21 | 4.8 (12.9) | 3.2  (7.7) | 2.9  (7.0) | 3.0  (7.0) | 3.8  (7.4) | 6.4  (13.0) | 6.8 (11.8) | 12.3 (21.9) | 2.9 (6.9) |
| fish^6^ | ≥30 | 21.0 (30.6) | 26.3  (27.5) | 21.8  (26.6) | 14.2  (22.6) | 19.6  (20.5) | 18.7  (25.2) | 14.9  (19.6) | 11.4  (21.8) | 15.3  (20.1) |

1. reference: Finnish Food Authority. Nutrition recommendations and food-based dietary guidelines. https://www.ruokavirasto.fi/en/foodstuffs/healthy-diet/nutrition-and-food-recommendations/ Accessed 08 Sept 2025. 2.Finnish food-based recommendations 2014, food groups with specific recommendations based on 10MJ (2400 kcal) daily intake converted to g/day/2500 kcal. 3. Consists of fresh, frozen, canned, and cooked vegetables, fresh, frozen, and canned fruit, and fresh and frozen berries. Recommendations reflect edible portion, while purchase behaviors include both edible and non-edible food parts (e.g., peels of fresh fruits). 4. Based on the most conservative estimate weekly raw meat recommendation of 700-750 g/week. Food purchase behaviors include both raw and cooked meats. 5. Based on daily pure alcohol intake no more than 10 g/d for women and 20 g/day for men. 6. Based on the most conservative estimate of recommendation for cooked fish consumption (i.e., 100-150 g of fish 2-3 times/week).

**Supplemental table 3.** Annual^1^ transition probabilities between food purchase behaviors from study participants (n=29,245)

|  | Health-oriented | | | | Discretionary-oriented | | | | Baby-oriented |
| --- | --- | --- | --- | --- | --- | --- | --- | --- | --- |
|  | Balanced | Home cook | Flexitarian | Planetary | Traditional | Convenience | SSB and snacks | Beer, sweets, and treats | Baby formula and foods |
| Balanced | **0.593** | 0.101 | 0.106 | 0.021 | 0.016 | 0.069 | 0.016 | 0.052 | 0.025 |
| Home cook | 0.075 | **0.692** | 0.060 | 0.011 | 0.062 | 0.059 | 0.011 | 0.014 | 0.017 |
| Flexitarian | 0.096 | 0.053 | **0.465** | 0.157 | 0.044 | 0.062 | 0.032 | 0.041 | 0.049 |
| Planetary | 0.042 | 0.022 | 0.288 | **0.444** | 0.024 | 0.041 | 0.045 | 0.056 | 0.038 |
| Traditional | 0.013 | 0.044 | 0.043 | 0.011 | **0.693** | 0.069 | 0.078 | 0.021 | 0.029 |
| Convenience | 0.041 | 0.049 | 0.050 | 0.014 | 0.071 | **0.539** | 0.075 | 0.126 | 0.036 |
| SSB and snacks | 0.019 | 0.013 | 0.038 | 0.028 | 0.087 | 0.107 | **0.553** | 0.121 | 0.035 |
| Beer, sweets, and treats | 0.069 | 0.021 | 0.051 | 0.029 | 0.032 | 0.224 | 0.133 | **0.408** | 0.033 |
| Baby formula and foods | 0.095 | 0.067 | 0.161 | 0.062 | 0.125 | **0.184** | 0.115 | 0.095 | 0.096 |

1. Transition probabilities over a 12-month interval were obtained by exponentiating the estimated transition intensity matrix.

Rows represent the starting state in a given month, and columns represent the state in the following month. SSB: Sugar-sweetened beverages. Diagonal values indicate the estimated probability of staying in the same state, while off-diagonal values indicate the probability of transitioning to a different state. The largest transition probability in each row is bolded.

**Supplemental figure 1** Bayesian Information Criterion for Hidden Markov Models reduced to 4-5 dimensions with 2-10 states. The 5‑dimension model with 9 states shows a distinct minimum and was therefore selected, noting that the 4‑dimension model with 10 states failed to converge and was set to NA.

**Supplemental figure 2** Overall prevalence of the most frequently observed food purchase behaviors of study participants (n=29,245) by categorized age, gender, household income, and household structure.

**Supplemental figure 3** Food purchase behaviors by gender (n=29,245)

Sequence index plots show estimated monthly states and transitions for participants from 9/2016-12/2018 grouped by gender sorted by last food purchase behavior (state) observed. SSB: Sugar-sweetened beverages.

a. Men, b. Women

**Supplemental figure 4** Food purchase behaviors by age (n=29,245)

Sequence index plots show estimated monthly states and transitions for participants from 9 9/2016-12/2018 grouped by decade-categorized age sorted by last food purchase behavior (state) observed. SSB: Sugar-sweetened beverages.

a. Under 30 years old, b. 30-39 year old, c. 40-49 year-old, d. 50-59 year-old, e. 60-69 year-old, f. 70 year-old and older

**Supplemental figure 5** Food purchase behaviors by household structure (n=29,245)

Sequence index plots show estimated monthly states and transitions for participants from 9/2016-12/2018 grouped by categorized household structures sorted by last food purchase behavior (state) observed. SSB: Sugar-sweetened beverages.

a. One adult households, b. Two adult households, c. One adult households with child(ren), d. Two adult households with child(ren), e. Other, including households with three or more adults or those with an unclassified family structure.

**Supplemental figure 6** Food purchase behaviors by household income (n=29,245)

Sequence index plots show estimated monthly states and transitions for participants from 9/2016-12/2018 grouped by categorized OECD-adjusted household income levels sorted by last food purchase behavior (state) observed. SSB: Sugar-sweetened beverages.

a. <1000 euros/month, b. 1000-1999 euros/month, c.2000-2999 euros/month, d. 3000-3999 euros/month, e. ≥4000 euros/month.
